# Hysteria with Fibroid Tumor of Uterus

**Published:** 1873-01

**Authors:** Benj. A. Fordyce

**Affiliations:** Union Springs, N. Y.


ART. II.—*Hysteria with Fibroid Tumor of Uterus.* By BENJ. A. FORDYCE, M. D., Union Springs, N. Y.

Read before the Central New York Medical Association.

Hysteria, independent of complication with organic disease, by its innumerable manifestations, is too well known to the medical profession to require description; but when existing cotemporaneous with grave organic lesion readily diagnosed and positively demonstrated, the inquiry may with propriety be pertinent, what dependence may the one have upon the other? Are the phenomena of Hysteria dependent upon the organic lesion? or are they primary and the change of structure a result?

That many cases of hysteria, which had been considered in the light of some nervous excitement, the pathology of which has been imperfectly understood, are found to be attended by organic lesions of important organs is now too well known to require additional proof.

A case to illustrate the foregoing statement is that of Miss. F., of Cayuga Co., native, age 34 years, a strong, thick-set woman of more than medium height, dark brown hair, dark eyes, ot fair complexion, weight 160. From the age of puberty she has been the unfortunate subject of convulsions at the menstrual period. No peculiarity different from the ordinary convulsions of hysterical subjects until some eight years since her menstrual discharge became hemorrhagic at each return, with alarming prostration. Convulsions continued, *more* severe, but not so frequent, sometimes passing over two or three months, then continuing in the most frightfully violent form two or three weeks.

About this time Dr. J. G. Richardson, formerly of Union Springs, now of Philadelphia Hospital, was her attending physician. Upon careful examination the doctor became satisfied that a fribroid tumor was developed in the substance of the uterus or its appendages. The propriety of removing it by an operation was considered, and I was called in consultation. The tumor was hard, inelastic, somewhat moveable, yet so intimately connected with the uterus that the idea was abandoned. We believed no operation could be performed without compromising the life of the patient. She consulted Dr. Briggs, of Auburn, who concurred in that opinion. Other surgeons were consulted, nearly all giving the same opinion. Afterwards, being always apparently ready for something heroic, she went to the Woman's Hospital of New York City, with intention of being operated upon, and was retained as a patient for several months, but returned with the tumor, no better satisfied than before. She then visited Dr. Potter, formerly of Geneva, now deceased, who gave her some encouragement that it could be removed without necessarily sacrificing her life. All her energies were expended in making preparation for an operation, but a new attack of convulsions wi'h hemorrhage partially frustrated her intentions and postponed her calculations. She was now (winter of 1870–'71) under the care of a Homeopathic practitioner, having some hysterical phenomena, such as retention of urine convulsions, etc. The doctor, becoming discouraged with the effects of his little pills and the great tax on his time, concluded to have an operation, the friends and patient all anxious for this distinction. He telegraphed to Dr. Potter for that object. Prior to the day appointed I was again consulted by the patient. At this time I made a careful examination of the uterus with Sim's Sound, and found that the enlargement had elongated the uterine canal to just five inches. This satisfied me that the substance of the uterus was included in the abnormal growth. I then gave my advice that if she was ready, prepared and wanted to die, to have the tumor removed. She wavered a little, but the doctor came agreeable to his appointment, and, after careful examination, though anxious to operate, would not renew his former assurance of safety to the patient. So (in my opinion) she was permitted to live for the benefit of science and to be the opprobrium of some other innocent doctor, the operation being indefinitely postponed. The Homeopathic doctor now became fairly disgusted with the patient, and asserted, with a verifying declaration, that "it would be better for her and her friends to have the operation performed now they were prepared for it, even if she did die," and refused longer to attend upon her.

We now arrive at a period in the history of the case when the most peculiar frieks of hysteria were to be enacted. A new medical attendant must be obtained. I was probably excused for the reason that I am not much of a favorite in this class of cases in that vicinity. A young physician was engaged, who, for care and fidelity to his patient, could not be excelled. The case progressed as usual for a time, when an attack of Dysphagia, attended with Aphonia, came on suddenly, to the alarm of physician and friends. The doctor informed me that she did not swallow a particle of anything or speak aloud for four weeks, although her brother, with whom she was living, who fully confirmed the doctor's statement s informed me that they prepared for her each night one and a half pints of rich oyster broth to wet her mouth with as it became dry! During this time she lost flesh, and at times was believed by her friends to be dead. The vital spark remained, however, and as suddenly as the attack came on, in an instant the functions were all restored.

This was followed in a few days with incessant and continued vomiting for several weeks, accompanied by retention of fæces. After exhausting the remedial powers treated of in the pharmacopia, the doctor resorted to croton oil, which, after administering some fifteen drops, had the desired effect.

But the most persistent and irremediable ailment to contend with was retention of urine, which became utterly intractable except to the use of the catheter. On the 8th July, 1871, the doctor commenced his operations with this instrument. No instructions that the doctor could give would avail anything to the patient. The doctor alone could satisfy to remove the secretions, and four times per day for sixteen months was he required to operate. The doctor tells me that at one time it was complicated with diabetes, when for several days he drew off five pints of clear limpid urine every *four* hours, by actual measure, making about thirty pounds passed in twenty-four hours. This excess gradully diminished and was followed by ascites, and this by pleuro-pneumonia, and then onychia, by which all the finger and toe nails were cast off twice during the last two years, with ulceration of matrix of nails.

Upon expressing his apprehensions to the patient that there might be stone in the bladder, in a few days he was amply rewarded by the appearance of gravel in the urine. This discharge was first observed on the 21st of June, '72. Here are some of the specimens which were claimed to have been passed at that time. The doctor showed them to me on the 23d of June. On examination with heat and chemicals, they were found to be just what they appear—slate gravel. I endeavored to satisfy the doctor that he was being imposed upon, but at first was unsuccessful. On the 26th of June, I was requested to see the patient with him. She was put under chloroform and examined with metalic catheter and no evidence of stone found in the bladder. Upon solicitation, I then dilated the urethra, by the method recommended by Dr.

More, successfully passing my finger into the bladder with the same result regarding stone, finding nothing abnormal. This, however, did not cure the evil, for now larger stone began to pass at each introduction of the catheter, or in the doctor's words, "mostly at night and early in the morning," some of these larger specimens I now exhibit to you. From the 26th of June to the 26th of November, the patient passed and the doctor removed three-fourths of a pound of stone of the appearance of those large specimens. I saw them weighed. She would have periods of fright, like a person in delirium tremens; although I should have stated that hypodermic injections of morphine were administered three or four times daily, sometimes one grain at a dose by weight.

Although this woman suffered and passed through all and more than I have related, she has worked at sewing and earned more than two hundred dollars last year, and labors daily when not too ill.

The extreme labor of quarryiny so many stone and pumping so much water began to tell seriously on the doctor's health, and he was taken ill in November, so as to be obliged to discontinue his attendance upon the patient. She then went to the Water Cure at Geneva, where, being subject to a change of discipline, or for some other reason not fully explained, the passage of stone from the urethra gradually diminished and in a few days ceased entirely. She was put under the care of female attendants and in a great measure regained several other impaired functions.

The menstrual flow of this patient was too frequent, being every two or three weeks, and excessive in quantity.

Several other cases have come under my observation of the same character of disease, in one of which similar phenomena were manifested for fourteen years, and at last terminated fatally. In this case I had an opportunity of diagnosing the organic lesion two years before, and of making a post mortem examination, when the uterus was found to be hypertrophied to the extent to weigh five pounds. Age 42.

In another case, age 31, married, the fribroid tumor originated from the posterior part of the fundus uteri, midway between the oviducts, and weighed three and one-fourth pounds. By its weight it had retroverted the uterus. Notwithstanding the malposition of the uterus, the woman became pregnant and went to full time. The tumor occupying the basin of the pelvis and being immovable, dilivery *via naturales* was impossible. After remaining in labor two days with no hope of relief from other aid, with the assistance of several physicians who were present, I delivered the woman of a living child by cæsaran section.

Hyeterical phenomena and all the unpleasant symptoms connected therewith were frequent in both of the latter cases.

*Query.*—Is not organic lesion of the uterus the cause of hysteria?

